# ﻿Description of *Chilearinus* Sharkey gen. nov. and status of Nearctic *Earinus* Wesmael, 1837 (Braconidae, Agathidinae) with the description of new species

**DOI:** 10.3897/zookeys.1099.81473

**Published:** 2022-05-03

**Authors:** Michael J. Sharkey, Austin Baker, Ramya Manjunath, Paul D. N. Hebert

**Affiliations:** 1 The Hymenoptera Institute, 116 Franklin Ave., Redlands, CA, 92373, USA The Hymenoptera Institute Redlands United States of America; 2 Department of Biological Sciences and Center for Biodiversity Research, University of Memphis, TN, USA University of Memphis Memphis United States of America; 3 Centre for Biodiversity Genomics, University of Guelph, Guelph, ON, Canada University of Guelph Guelph Canada

**Keywords:** Accelerated taxonomy, BIN code, COI barcode Hymenoptera, COI DNA barcode, conservation, Ichneumonoidea

## Abstract

The Neotropical members formerly included in *Earinus* Wesmael, 1837 are transferred to a new genus, *Chilearinus* Sharkey **gen. nov.** Presently three Nearctic species of *Earinus* are recognized, i.e., *Earinuserythropoda* Cameron, 1887, *Earinuslimitaris* Say,1835, and *Earinuszeirapherae* Walley, 1935, and these are retained in *Earinus*. *Earinuschubuquensis* Berta, 2000 and *Earinusscitus* Enderlein, 1920 are transferred to *Chilearinus*, i.e., *C.chubuquensis*, and *C.scitus*, **comb. nov.** One other species is transferred to *Chilearinus*, i.e., *Microgasterrubricollis* Spinola, 1851, *Chilearinusrubricollis*, **comb. nov.** Two other Neotropical species, *Earinushubrechtae* Braet, 2002 and *Earinusbourguignoni* Braet, 2002 were described under the genus *Earinus* but are here transferred to *Lytopylus*, *L.hubrechtae*, and *L.bourguignoni***comb. nov.** Two new species of *Chilearinus* are described, *C.covidchronos* and *C.janbert***spp. nov.** The status of *Agathislaevithorax* Spinola,1851, *Agathisrubricata* Spinola,1851, and *Agathisareolata* Spinola, 1851 is discussed. A neotype is designated for *Earinuslimitaris* (Say, 1835) and diagnosed with a COI barcode. *Earinusaustinbakeri* and *Earinuswalleyi***spp. nov.** are described. The status of both *Earinus* and *Chilearinus* in the Americas is discussed. A revised key to the genera of Agathidinae of the Americas is presented.

## ﻿Introduction

Neotropical species formerly included in *Earinus* Wesmael, 1837 are transferred to a new genus, *Chilearinus* Sharkey gen. nov. Presently three Nearctic species of *Earinus* are recognized, i.e., *Earinuserythropoda* Cameron, 1887, *Earinuslimitaris* Say,1835, and *Earinuszeirapherae* Walley, 1935, and these are retained in *Earinus*. *Earinuschubuquensis* Berta, 2000 and *Earinusscitus* Enderlein, 1920 are transferred to *Chilearinus*, i.e., *C.chubuquensis* and *C.scitus*, comb. nov. One other species is transferred to *Chilearinus*, i.e., *Microgasterrubricollis* Spinola, 1851, *Chilearinusrubricollis*, comb. nov. Two other Neotropical species, *Earinushubrechtae* Braet, 2002, and *Earinusbourguignoni* Braet, 2002 were described under the genus *Earinus* but are here transferred to *Lytopylus*, *L.hubrechtae*, and *L.bourguignoni* comb. nov. Two new species of *Chilearinus* are described, *C.covidchronos* and *C.janbert* spp. nov. The status of *Agathislaevithorax* Spinola,1851, *Agathisrubricata* Spinola,1851, and *Agathisareolata* Spinola, 1851 is discussed. A neotype is designated for *Earinuslimitaris* (Say, 1835) and diagnosed with a COI barcode. *Earinusaustinbakeri* and *Earinuswalleyi* spp. nov. are described. The status of both *Earinus* and *Chilearinus* in the Americas is discussed. A revised key to the genera of Agathidinae of the Americas is presented.

## ﻿Methods

### ﻿DNA extraction and sequencing

Molecular work was carried out at the CBG using standard protocols. A leg from each frozen-then-oven-dried specimen was destructively sampled for DNA extraction using a glass fiber protocol (Ivanova et al. 2006). Extracted DNA was amplified for a 658 bp region near the 5′ terminus of the cytochrome *c* oxidase subunit I (COI) gene using standard insect primers LepF1 (5′-ATTCAACCAATCATAAAGATATTGG-3′) and LepR1 (5′-TAAACTTCTGGATGTCCAAAAAATCA-3′) (Ivanova and Grainger 2007). If initial amplification failed, additional PCRs were conducted following established protocols using internal primer pairs: LepF1–C113R (130 bp) or LepF1–C_ANTMR1D (307 bp) and MLepF1–LepR1 (407 bp) to generate shorter overlapping sequences. Most amplicons were Sanger sequenced, but some recent specimens were analyzed on SEQUEL.

The BOLD database can be used to identify specimens using the following steps: (1) navigate to the identification tab of the BOLD Systems database (http://www.boldsystems.org/index.php/IDS_OpenIdEngine); (2) paste the COI sequence of the query organism (in forward orientation) into the query box and search against the appropriate library (e.g., All Barcode Records on BOLD, Species Level Barcode Records, etc.); (3) the search results page shows the top hits based on percentage similarity starting with the closest matches (This page also provides additional information to help verify the identity of a match, such as links to the BIN where specimen data, including images, can be found, a distribution map, and a tree-based identification tool); (4) use the Tree-Based Identification button to generate a neighbor-joining tree and find the query taxon (name in red). This allows you to visualize how distant the query sequence is from the closest matches.

## ﻿Taxonomic account

### ﻿ Chilearinus

Taxon classificationAnimaliaHymenopteraBraconidae

Sharkey gen. nov.

3E25AD77-2F67-5C51-B86E-AEDE5CA7DB52

http://zoobank.org/82CEAEE1-8CDB-48DD-B79F-1B59F8CF74A1

#### Type species.

*Chilearinusjanbert* Sharkey, sp. nov.

#### Etymology.

A conjunction of Chile, where 90% of the species are likely to be found, and *Earinus*, a reference to the probable sister group of the species, based on preliminary analyses. The genus is masculine.

#### Diagnosis.

Notauli absent; hind coxal cavities open; tarsal claws with basal lobes; second submarginal cell quadrate, never petiolate; foretibia lacking sclerotized spines/pegs; hind wing Cub strong and emanating from an angle on the basal cell. Most similar morphologically to *Earinus* and *Lytopylus*. *Earinus* and *Chilearinus* do not have overlapping distributions. The former is restricted to the Nearctic and the latter to the Neotropics; therefore, there is little chance of confusing the two. Nonetheless, the lack of pegs on the foretibia of members of *Chilearinus* and the morphological characters given in the key (below) can also be employed to differentiate them. Members of *Lytopylus* differ most significantly in that they lack vein Cub in the hind wing. See couplet 25 in the key below.

#### Description.

***Head*.** Lateral carina on frons (as found in members of *Alabagrus*) absent; interantennal space slightly raised above antennal sockets; gena not extended ventroposteriorly into sharp prominence; mandible dorsoventrally flattened (twisted); labial palpus with 4 segments, third segment slightly more than ½ length of apical segment. ***Mesosoma*.** Propleuron lacking a sharp bump; notauli absent; mesoscutum smooth with a median pit (presumably a remnant of notauli), postscutellar depression absent; propodeum mostly smooth, sometimes with weak smooth sculpture medially; sclerite between hind coxal cavities and metasomal foramen absent. Precoxal groove absent or smooth and weakly impressed. ***Legs*.** Foretibia lacking dull pegs (unlike *Earinus*); mid- and hind tibia with blunt apical or preapical pegs; all tarsal claws with a rounded basal lobe. ***Wings*.** Forewing RS+Ma vein mostly present but not usually completely tubular; second submarginal cell large, quadrate and usually (perhaps always) higher than long; RS of forewing complete to wing margin; hind wing r and r-m cross veins absent; hind wing vein Cub strong and emanating from an angle on the basal cell. ***Metasoma*.** First median tergite smooth, longer than apical width, lateral longitudinal carina absent or weak and short; remaining terga smooth; ovipositor ranging from as long as the body to twice the length of the body, but this is based on small sample of a few dozen species.

#### Biology.

Unknown.

#### Diversity and distribution.

This is a species-rich genus with hundreds of species, based on specimens identified by MS. It is widespread in Chile and southern Argentina. A few species are found at high altitudes as far north as Ecuador and Colombia.

#### Notes.

Sharkey (1997) included members of what are now *Chilearinus* in a broader concept of *Earinus*. Spinola (1851) described three species of Agathidinae from Chile. Since members of *Chilearinus* are by far the most species-rich of Chilean agathidines, and since his descriptions do not contradict membership in the genus, these species are probably members of *Chilearinus*, i.e., *Agathislaevithorax*, *Agathisrubricata*, and *Agathisareolata*. They certainly are not members of *Agathis* since this genus does not extend into the southern regions of South America. These specimens should be in the Hymenoptera collection of Maximilian Spinola whose collection is housed in the Museo Regionale di Scienze Naturali (MRSN) in Turin (Torino). One of us (MS) could not locate these specimens during a visit to MSRN in 1985, but a specimen of *Chilearinus*, *Microgasterrubricollis* Spinola, 1851, was present. *Microgaster* may seem an odd place for placement of what we now consider an agathidine, but such was the classification at the time. It is clear from the following that Spinola knew the species was closely related to *Earinus*, “Este *Microgastro* habria pertenecido á [sic] la primera seccion del *G. Microdus*, N. V. Es., y al sub-género *Earinus* Wesm.” (Spinola 1851: 34).

It is almost pointless to present a morphological key to the five recognized species of *Chilearinus* as they represent just five species out of hundreds. Many undescribed species will undoubtedly key to these named species. The only way to handle species-rich undocumented genera such as *Chilearinus* is to include COI barcode data in the diagnoses. We know this diagnostic is sufficient to differentiate all but a few species of Agathidinae (Sharkey et al. 2018). Nonetheless, despite the absurdity, a key is presented below to mollify critics (e.g., Zamani et al. 2020).

### ﻿Key to the few described species of *Chilearinus*

**Table:** 

1	Forewing with two yellow bands	***C.scitus***
–	Forewing evenly colored, weakly infuscate	**2**
2	Mesonotum orange	***C.rubricollis***
–	Mesonotum black	**3**
3	Hind femur entirely yellow except extreme apex dorsally	***C.janbert***
–	Hind femur mostly or entirely black	**4**
4	Hind femur black except extreme apex yellow	***C.chubuquensis***
–	Hind femur entirely black	***C.covidchronos***

### ﻿ Chilearinus covidchronos

Taxon classificationAnimaliaHymenopteraBraconidae

Sharkey sp. nov.

037D9453-A2E9-514F-BE98-5E9543608FF4

http://zoobank.org/67B17FE2-0DD1-4E44-A862-A5E3275E3D8D

Fig. 1

#### Holotype.

♀, Chile, Región IX, PN Nahualbuta, 37.809°S, 73.016°W, 3680' [1122 m], 9–12.i.2000, Malaise trap, Webb and Yeates (Canadian National Collection).

#### Diagnosis.

COI barcode. BOLD sample ID H1145. BOLD BIN code BOLD:AAV0870. GenBank Accession Code OL702761.

AATTTTATATTTTATATTTGGAATTTGATCGGGAATTTTAGGTTTATCAATAAGTTTAATTATTCGAATAGAATTAAGAGTAGGGGGTAATTTTATTGGTAATGATCAAATTTATAATAGAATTGTNGCTGCTCATGCTTTTATTATAATTTTTTTTATAGTTATACCAATTATAATTGGAGGATTTGGAAATTGATTAATTCCATTAATATTGGGGGGGCCAGATATAGCTTTCCCTCGAATAAATAATATAAGATTTTGATTATTAATTCCTTCATTATTATTATTAATTTTAAGGTCTTTAATTAATGTTGGGGTAGGTACTGGATGAACTGTTTATCCTCCTTTATCATTAAATATAAGTCATAGTGGTATATCTGTAGATTTAGCTATTTTTTCTTTACATATTGCTGGAATTTCTTCAATTATAGGTGCTATAAATTTTATTACAACTATTTTAAATATGTGAATAATTAATATTAAAATTGATAAAATACCTTTATTAGTTTGATCAATTTTAATTACGGCAATTTTATTATTATTATCTTTGCCAGTTTTAGCTGGAGCTATTACTATATTATTAACAGATCGTAATTTAAATACTAGATTTTTTGATCCTTCTGGAGGAGGAGATCCAATTTTATATCAACATTTATTT

#### Morphological diagnosis.

See key.

#### Paratypes.

None.

#### Etymology.

Named in acknowledgment of the covid pandemic occurring during the production of this manuscript.

**Figure 1.**
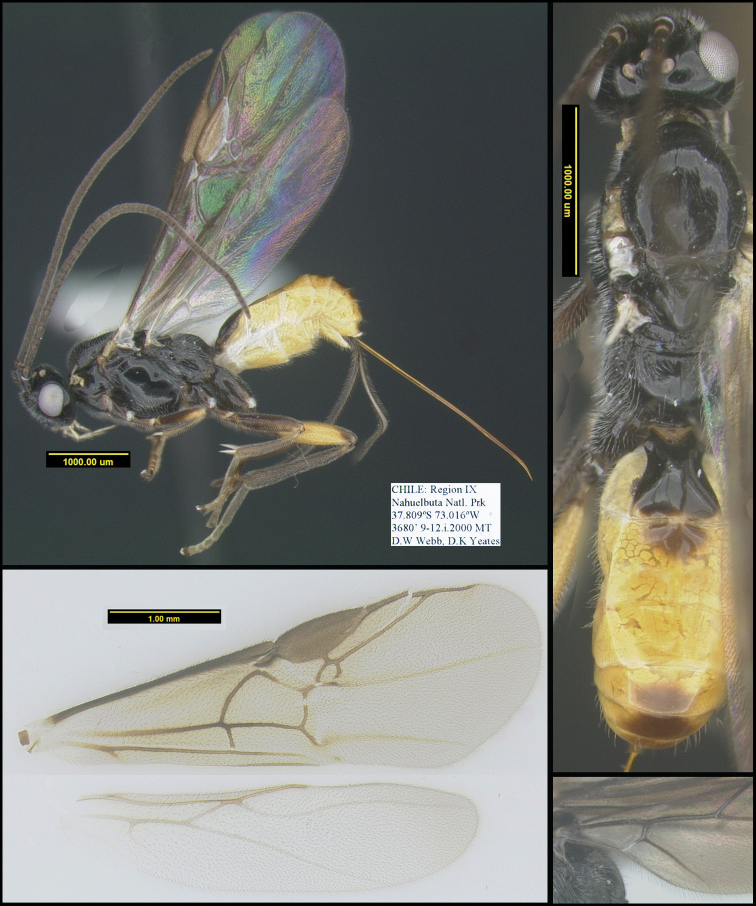
*Chilearinuscovidchronos* Sharkey, sp. nov., holotype.

### ﻿ Chilearinus janbert

Taxon classificationAnimaliaHymenopteraBraconidae

Sharkey sp. nov.

AB9C892C-E5CA-5F6A-BE83-F0ED72EAEB87

http://zoobank.org/AF4C4A3B-EBD8-4305-AF39-DC9176C868A8

Fig. 2

#### Holotype.

♀, Chile, Región IX, PN Nahualbuta, 37.493°S, 72.582°W, 1168 m, 8.ii.2005, Heraty, (Canadian National Collection).

#### Diagnosis.

COI barcode. BOLD sample ID H12114. BOLD BIN: BOLD:AEM7846. GenBank Accession Code OL702760.

TTTTAGGATTATCAATAAGTTTAATTATTCGAATAGAATTAAGAGTAGGTGGTAATTTTATTGGTAATGATCAAATTTATAATAGGATTGTNACTGCTCATGCTTTTATTATAATTTTTTTTATAGTTATACCAATTATAATTGGAGGATTTGGAAATTGATTAATTCCATTAATATTAGGGGGTCCAGATATAGCCTTCCCTCGAATAAATAATATAAGATTTTGATTATTAATTCCTTCATTATTATTATTAATTTTAAGATCTTTAATTAATGTTGGAGTAGGTACTGGATGAACTGTTTATCCTCCTTTATCATTAAATATAAGTCATAGTGGTATATCTGTAGATTTGGCTATTTTTTCTTTACATATTGCTGGAATTTCTTCAATTATAGGGGCTATAAATTTTATTACAACTATTTTAAATATATGAATAATTAATATTAAAATTGATAAAATACCTTTATTAGTTTGATCAATTTTGATTACAGCAATTTTATTATTATTATCTTTACCAGTTTTAGCTGGGGCTATTACTATATTATTAACAGATCGTAATTTAAATACTAGATTTTTTGATCCTTCTGGAGGGGGAGATCCAATTTTATATCAACATTTATTTTGATTTTT

#### Morphological diagnosis.

See key.

#### Paratypes.

None.

#### Etymology.

A conjunction of Paul Hebert and Dan Janzen in recognition of their enormous contributions towards the conservation of nature.

### ﻿ Earinus

Taxon classificationAnimaliaHymenopteraBraconidae

Wesmael, 1837

3EB02370-41B3-561B-B842-5117B904EE03

#### Note.

In the Americas, there are three previously recognized species of *Earinus*, i.e., *E.erythropoda* Cameron, 1887, *E.limitaris* (Say, 1835), and *E.zeirapherae* Walley, 1935, and here we describe two more, *Earinusaustinbakeri* sp. nov. and *Earinuswalleyi* sp. nov. In the Nearctic, *Earinus* is common and widespread with the southernmost record being the sole recognized specimen of *E.erythropoda* from northern Sonora state, Mexico. *Earinus* differs from *Chilearinus* in the possession of pegs/spines in the foretibia and the characters given in the key.

**Figure 2.**
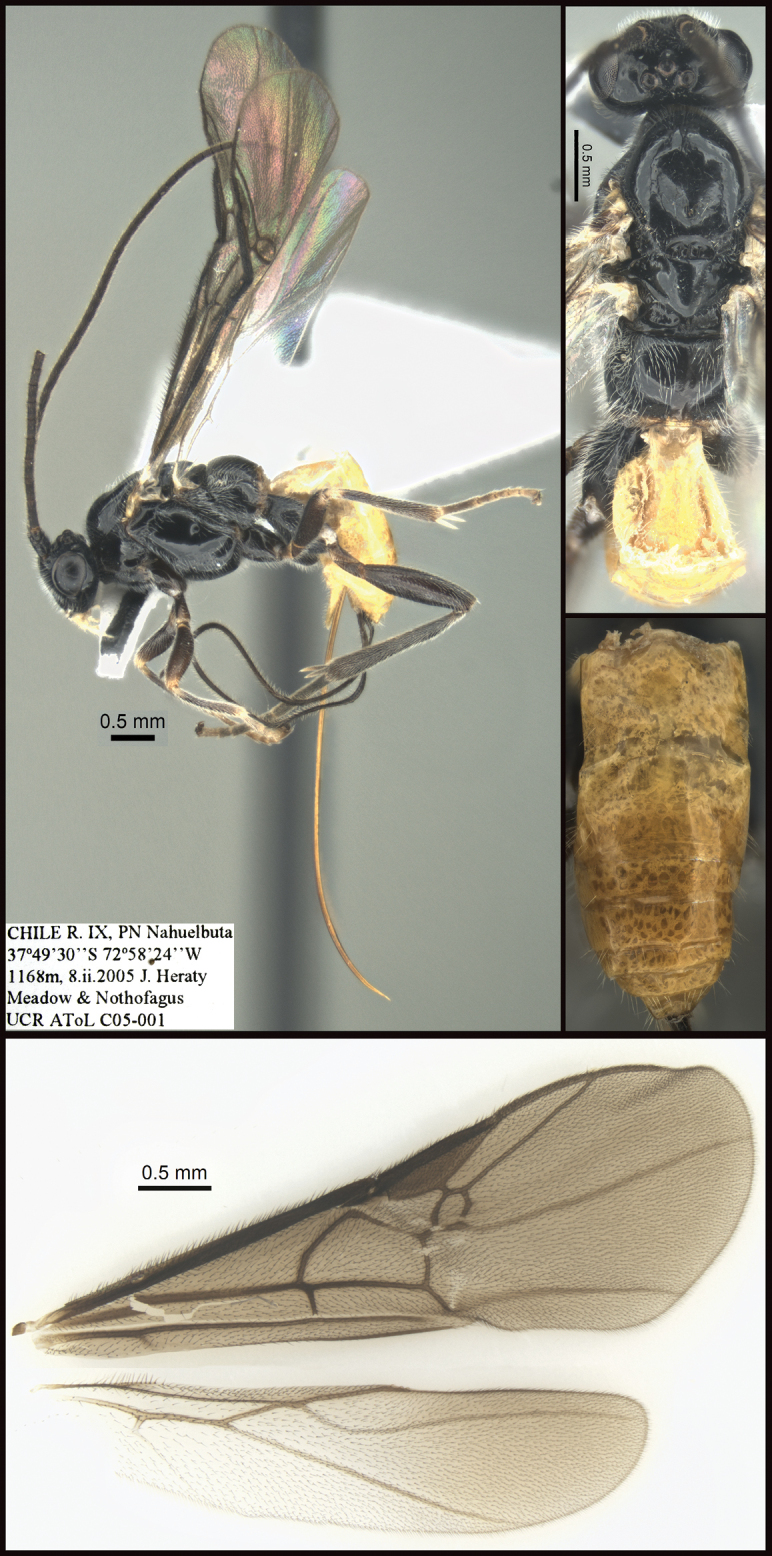
*Chilearinusjanbert* Sharkey, sp. nov., holotype.

Based on the collection in the Hymenoptera Institute (MS’s personal collection, which will eventually be deposited in the CNC) and borrowed specimens, there are probably between eight and 12 species in the Nearctic region. They are extremely similar in color, but there are obvious differences among specimens in body dimensions, degree of punctation, color of the hind coxae, ocellar configuration, ovipositor length, length and density of setae on the ovipositor sheath, and dimensions of the first metasomal tergum. Unfortunately, these are not sufficient to allow confident delineation of species limits. For example, the differences in the key between *E.limitaris* and *E.erythropoda* are trivial. There are numerous specimens scattered over the Nearctic region that will key to *E.erythropoda*, but they might all be *E.limitaris*, or the two nominal species may be conspecific, or there may be multiple cryptic species. Likewise, there are probably a number of undescribed Nearctic species that will key to either *E.zeirapherae* or *E.austinbakeri*. In other words, the key is sufficient to discriminate among the barcoded species and *E.zeirapherae* but not among these and the undescribed species. The key is presented in part to satisfy the code of Zoological Nomenclature to act as a diagnosis for *E.austinbakeri* and *E.walleyi*. Only dense sampling of COI barcodes and perhaps other genes will supply the information necessary to delimit Nearctic *Earinus* species.

### ﻿Key to the species of *Earinus* of North America

**Table:** 

1	**A** Mid- and hind coxae slightly (**A**) to distinctly (**AA**) melanic, darker than their respective femora	***E.zeirapherae***
–	**B** Mid- and hind coxae mostly or entirely pale (yellow to orange) concolorous with their respective femora	**2**
	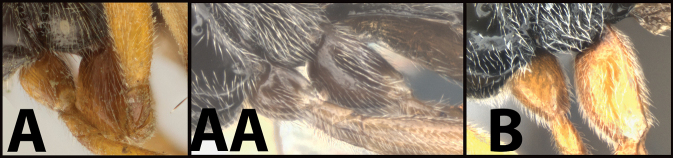	
2(1)	**A** Face distinctly punctate. Body length > 6 mm (average = 6.8 mm.)	**3**
–	**B** Face mostly smooth with shallow punctation. Body length < 6 mm (average = 5.3 mm)	**5**
	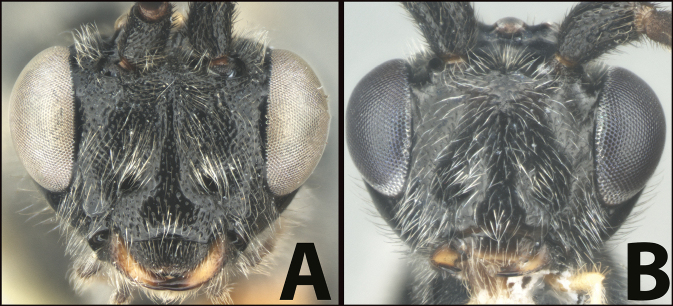	
3(2)	**A** Facial punctures deeper and wider	***E.limitaris* variation, or perhaps *E.* sp. nov.**
–	**B** Facial punctures shallower and narrower	**4**
	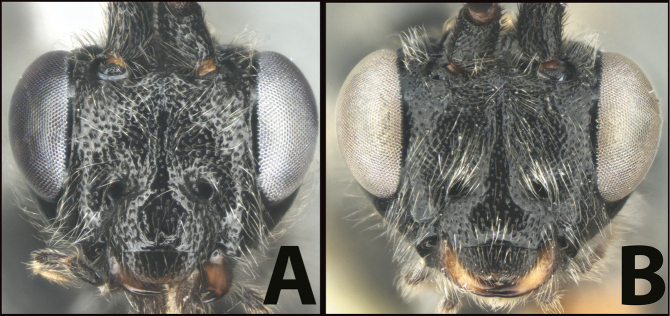	
4(3)	**A** Distance between lateral ocelli longer than distance between lateral ocellus and eye. **AA** Second submarginal cell lacking distinct 2RS2 vein	***E.limitaris***
–	**B** Distance between lateral ocelli equidistant or shorter than distance between lateral ocellus and eye. **BB** Second submarginal cell with distinct 2RS2 vein	***E.erythropoda***.[**Line drawings modified from Berta (2000)**]
	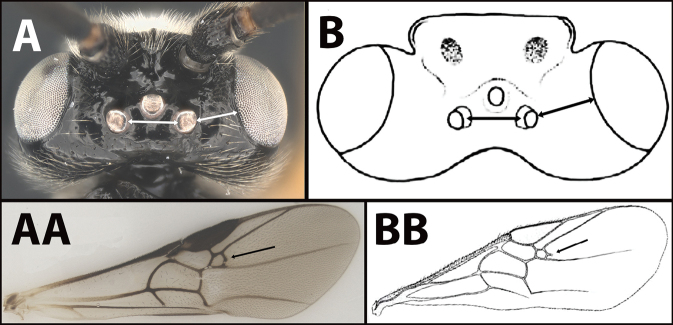	
5(2)	**A** First metasomal tergum relatively longer and slimmer, distinctly longer than wide	***E.austinbakeri***
–	**B** First metasomal tergum relatively shorter and broader, about as long as wide	***E.walleyi***
	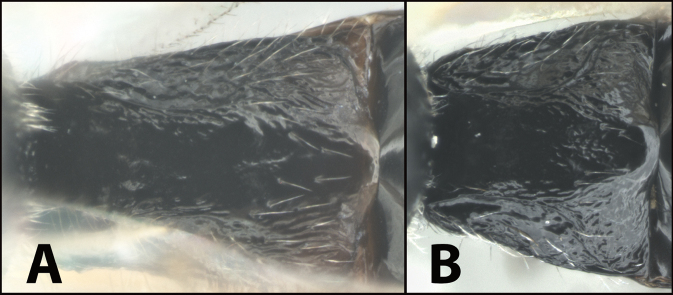	

**Figure 3.**
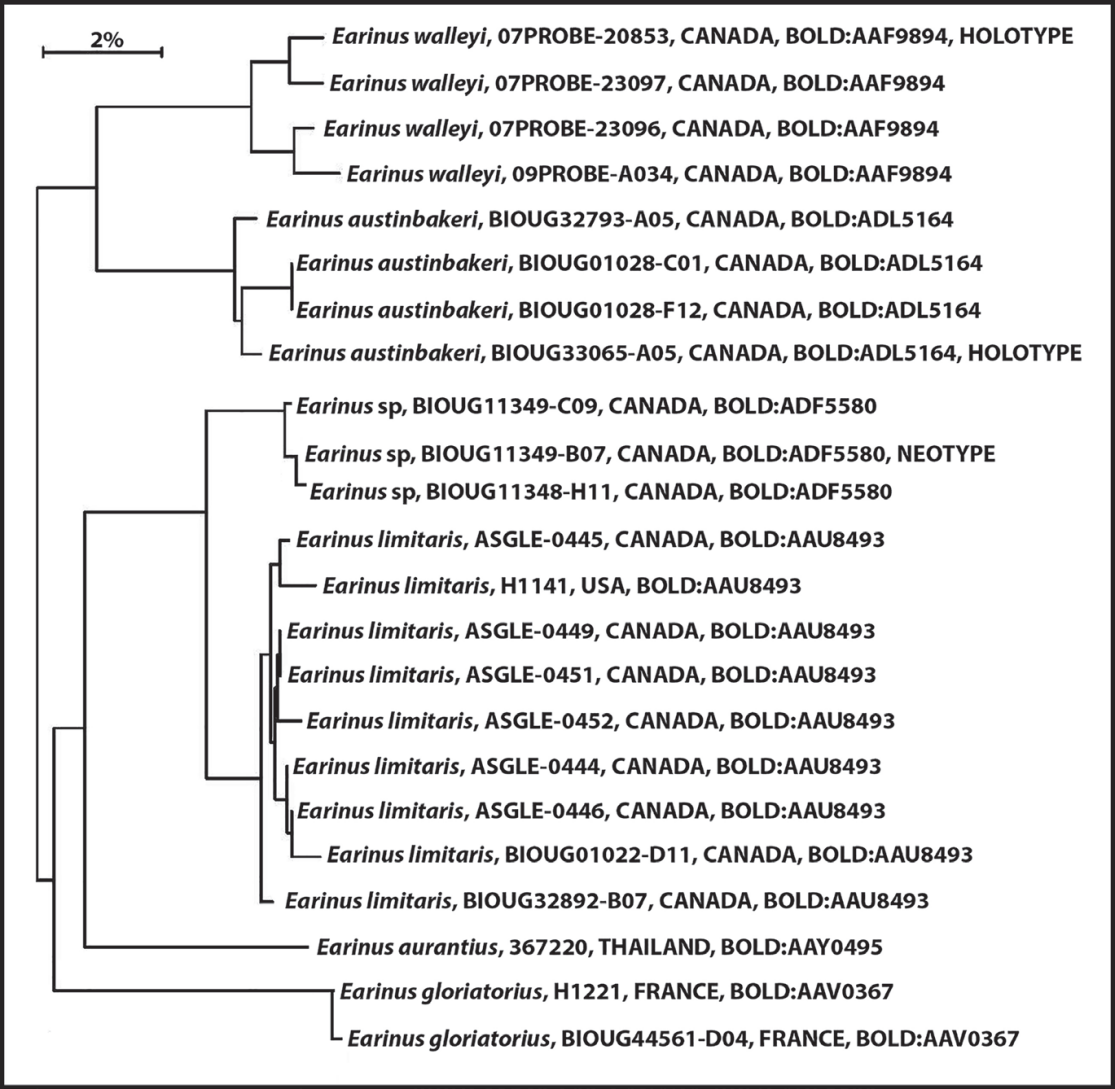
Neighbor joining tree of specimens of *Earinus* on BOLD with unique barcodes over 400 base pairs long (BOLD accessed 2022-1-20).

### ﻿ Earinus austinbakeri

Taxon classificationAnimaliaHymenopteraBraconidae

Sharkey sp. nov.

107889AA-0F66-5D06-A568-CA774826DA26

http://zoobank.org/D169A981-8A48-4E53-B1D1-CB072D898147

Figs 4 , 5

#### Holotype.

♀, Canada, Ontario, Ferris Provincial Park, 44.2829°N, 77.7963°W, 131 m, 05–20.Jun.2014 (Canadian National Collection). BOLD sample ID BIOUG33065-A05, BOLD BIN code BOLD:ADL5164. GenBank Accession Code OM158425.

#### Diagnosis.

Consensus barcode based on four specimens.

ATTTTATATTTTATATTTGGGATTTGATCYGGAATTGTGGGKTTATCAATAAGTTTAATTATTCGTATGGARTTAAGAGTAGGGGGBAATTTAATTGGKAATGATCAAATTTATAATAGTATTGTTACTGCTCATGCATTTATTATAATTTTTTTTATAGTTATRCCAATTATAATTGGTGGGTTTGGTAATTGGTTAATTCCTTTAATATTAGGRGGTCCCGATATRGCTTTCCCTCGAATGAAYAATATAAGRTTTTGATTATTAATTCCTTCTTTATTATTATTAATTTTAAGATCTTTAATTAATATTGGGGTTGGAACTGGTTGAACGGTYTATCCTCCTTTATCATTRAATATAAGTCATAGTGGTATATCTGTTGATTTGGCTATTTTYTCTTTACATATTGCGGGRATTTCTTCTATTATAGGGGCAATAAATTTTATTACTACTATTTTAAATATATGAATAATAAATATTAAAGTTGATAAAATGTCTTTATTRATTTGATCAATTTTAATTACTGCTATTTTATTATTATTATCTTTACCTGTTTTAGCRGGRGCAATTACTATATTATTAACAGATCGTAATTTAAATACAAGATTTTTTGATCCTTCTGGAGGTGGGGATCCAATTTTATATCAACATTTATTT

#### Morphological diagnosis.

Very similar to *E.austinbakeri* but differing by the characters given in the key as well as having the ovipositor sheath more setose. The COI barcodes of the two species differ by 6.29% (*p*-distance), reinforcing the conclusion that they are different species.

**Figure 4.**
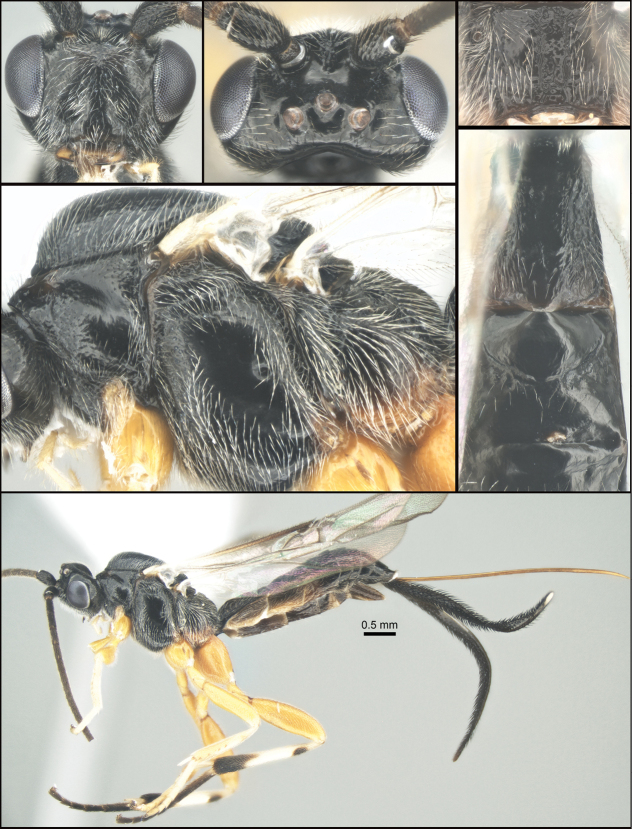
*Earinusaustinbakeri* Sharkey, sp. nov., holotype.

#### Paratypes.

BIOUG01028-C01, BIOUG01028-F12, BIOUG32793-A05. These are sample IDs; the data for these specimens can be found by searching for these codes on BOLD (http://www.boldsystems.org).

**Figure 5.**
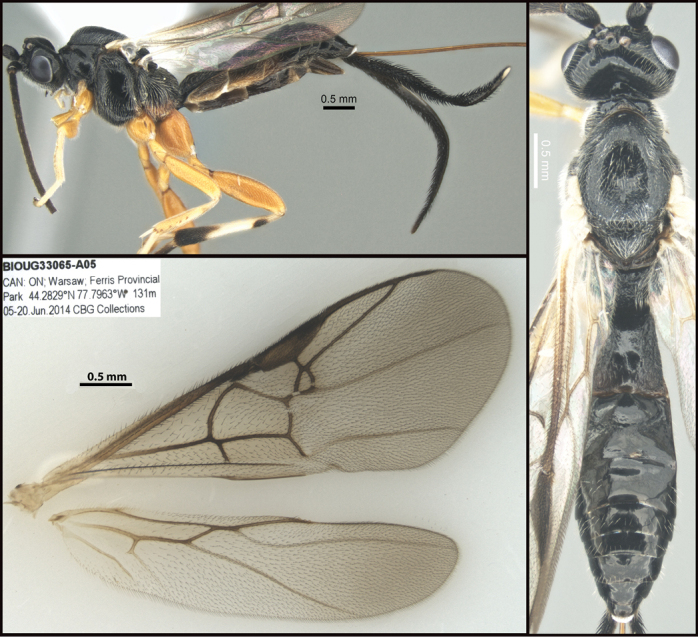
*Earinusaustinbakeri* Sharkey, sp. nov., holotype.

#### Distribution.

The holotype and paratypes were found at two localities just north and northeast of Lake Ontario. This species may be widespread throughout the eastern USA as far south as the Carolinas.

#### Etymology.

Named in honor Austin Baker, hymenopterist extraordinaire.

### ﻿ Earinus erythropoda

Taxon classificationAnimaliaHymenopteraBraconidae

Cameron, 1887

95DAC2F1-BA5F-506D-971D-F212E15AC226

#### Holotype.

♀, “N. Sonora, Mexico, Morrison” (British Museum Natural BM3c893, viewed).

#### Notes.

The sole identified specimen is the holotype. It differs little from many specimens that are widespread in the United States. It could be that they all belong to *E.limitaris*, or several more species may have similar morphologies. COI barcode data are needed. Several line drawings, modified from Berta (2000), are included in the key and others are in Berta’s (2000) treatment.

### ﻿ Earinus limitaris

Taxon classificationAnimaliaHymenopteraBraconidae

(Say, 1835)

EAE38190-F29E-5D79-A78E-22DCB293E7E9

Figs 6 , 7

Bassus limitaris Say, 1835.

#### Neotype.

♂, USA, West Virginia, Hardy County, 3 mi. NE Mathias, 38°55'N, 78°49'W, 30.viii–19.ix.2005 (Canadian National Collection). BOLD sample ID H1141. BOLD BIN code BOLD:AAU8493. GenBank Accession Code OM237775.

#### Diagnosis.

Consensus COI barcode based on 9 specimens.

AATTTTATATTTTATATTTGGAATTTGATCAGGAATTTTAGGTTTATCAATAAGATTAATTATTCGAATAGAATTAAGDATAGGTGGTAATTTRATTGGTAATGATCAAATTTATAATAGTGTTGTTYCTGCTCATGCTTTTATTATAATTTTTTTTATAGTTATACCAATTATGATTGGRGGRTTTGGRAATTGATTAGTTCCTTTAATATTGGGRGGTCCTGATATAGCTTTYCCTCGAATAAATAATATAAGATTTTGATTATTAATTCCTTCTTTATTATTATTAATTTTGAGTTCTTTAATTAATATTGGGGTRGGGACTGGKTGAACAGTTTATCCTCCRTTATCTTTAAATATAAGRCATAGTGGAATATCAGTTGATTTAGCTATTTTTTCATTACATATYGCAGGAATTTCTTCAATTATAGGGGCAATAAATTTTATTACTACTATYATAAATATATGAATAATAAATATTAAAATTGATAAAATACCTTTATTAGTTTGATCAATTTTAATTACTGCTATTTTATTATTATTATCATTRCCAGTTTTAGCTGGRGCAATTACTATATTATTAACAGATCGAAATTTRAATACAAGATTTTTTGATCCTTCTGGAGGGGGGGATCCAATTTTATATCAACATTTATTT

#### Morphological diagnosis.

See key.

#### Other specimens with barcode data.

ASGLE-0444, ASGLE-0446, ASGLE-0449, ASGLE-0451, ASGLE-0452, ASGLE-0445, BIOUG01022-D11, BIOUG32892-B07. These are sample IDs; data on them can be found by searching for these codes on BOLD (http://www.boldsystems.org).

#### Biology.

The following are listed as hosts of *E.limitaris* by Yu et al. (2016); all belong to Noctuidae: *Egiradolosa*, *Enargiadecolor*, *Homoglaeahircina*, *Ipimorphapleonectusa*, and *Orthosiahibisci*. Because there are probably a number of cryptic species in *E.limitaris*, these records need confirmation.

#### Notes.

There are 15 specimens from one locality in Quebec that are in a different BIN (BOLD:ADF5580) which differs by only 2.54% (*p*-distance) from *E.limitaris* (*Earinus* sp. in Fig. 3). Because of the small distance between these two BINs, we refrain from describing this BIN as a new species but suggest that it may be a distinct species. Broader geographic sampling is required to clarify the significance of this barcode split.

Like many of Say’s types, the type of *B.limitaris* is lost (Muesebeck 1927).

The following is from Say’s original description.

“*B*[*assus*] *limitaris*. Black; feet honey-yellow.

Inhabits Missouri and Indiana.

Body black: palpi white: thorax longitudinally indented behind the middle: wings nearly hyaline, at base yellowish; nervures fuscous; stigma large; first cubital cell complete; second rather large, quadrangular: radial cellule also rather large: feet honey-yellow; posterior pair of tibiae whitish, their tips and annulus near the base black; posterior pair of tarsi black.

**Figure 6.**
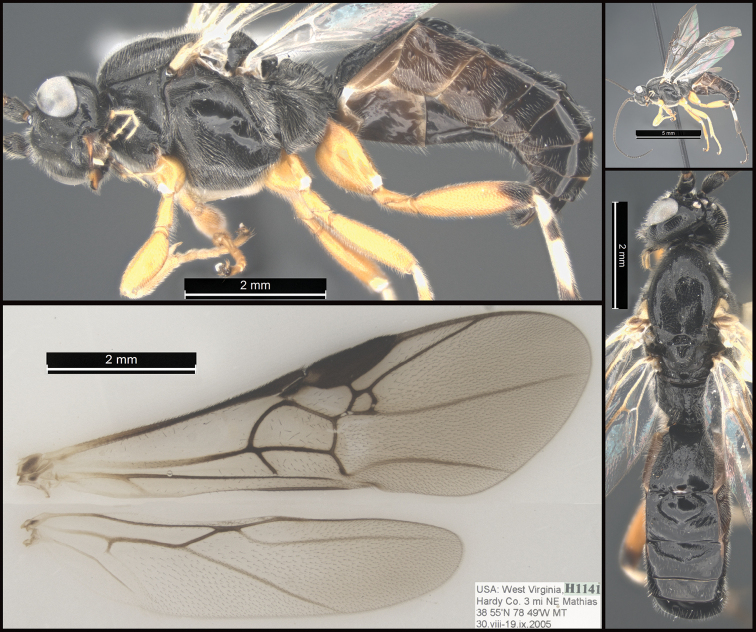
*Earinuslimitaris*, neotype.

Length seven twentieths of an inch.

Var. a. Maxillary palpi, first joint black.

♀ Oviduct hairy, decurved, somewhat robust.”

Except for the body length, this description is consistent with all of the estimated 8–12 Nearctic species of *Earinus*. We have a number of specimens of what we believe to be *E.limitaris*. The neotype was selected because it is geographically closest to the two specimens included in Say’s (1835) original description, despite the fact that it is a male.

**Figure 7.**
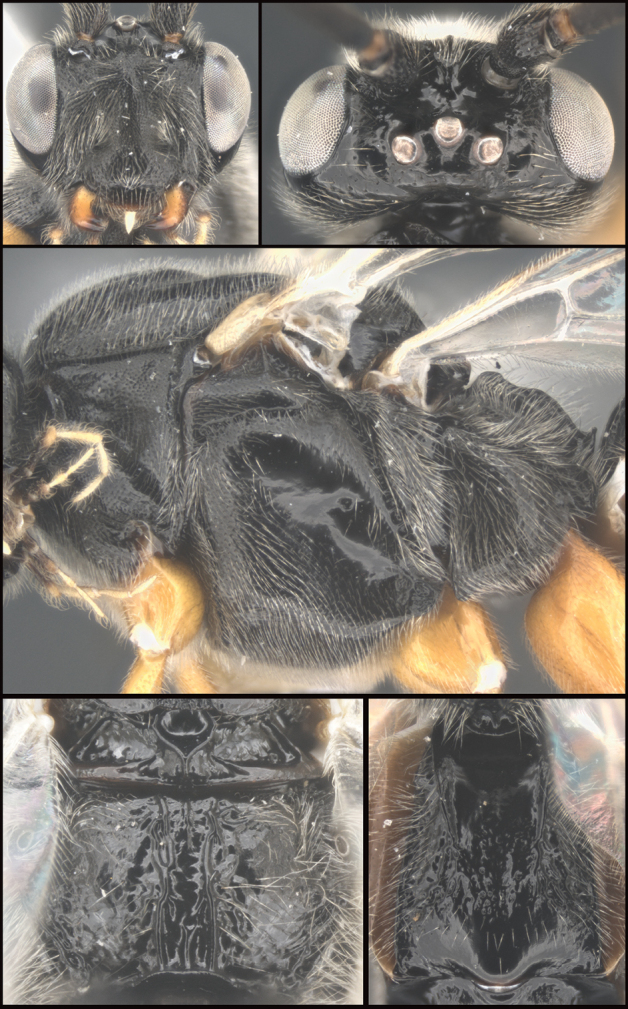
*Earinuslimitaris*, neotype.

#### Distribution.

Unknown, except for barcoded specimens (West Virginia, southern Ontario), as well as either Missouri or Indiana, or both. It is unknown if Say’s (1835) two specimens are conspecific. Based on specimens that one of us (MS) recently viewed, this species is probably widespread across southern Canada and northern United States, extending south as far as southern New Mexico (presumably at high altitudes) in the west and South Carolina in the east. The holotype of *E.erythropoda* may also belong here, which would extend the distribution into northern Sonora state, Mexico.

### ﻿ Earinus walleyi

Taxon classificationAnimaliaHymenopteraBraconidae

Sharkey sp. nov.

8DA974A7-E3F6-5E6F-A2B2-FF42AC77EBAA

http://zoobank.org/BDFBEADA-2082-46A5-B648-EB181E09CBB5

Figs 8 , 9

#### Holotype.

♀, Canada, Manitoba, Churchill pump house, 15 km S Churchill, Goose Creek Road, 58.3734°N, 94.1342°W, 3–7.vii.2007, Malaise trap (Canadian National Collection). BOLD sample ID. 07PROBE-20853, BOLD BIN code BOLD:AAF9894. GenBank Accession Code FJ413805.

#### Diagnosis.

Consensus barcode based on four specimens.

TATTTTATATTTTATATTTGGAATTTGATCAGGTATTGTAGGTTTATCAATAAGATTAATTATTCGAATGGAATTAAGAGTGGGRGGTAATTTAATTGGRAATGATCAAATTTATAATAGTATTGTTACTGCTCATGCTTTTATTATAATTTTTTTTATAGTTATACCTATTATAATTGGGGGRTTTGGTAATTGATTARTCCCATTAATATTGGGAGGTCCTGATATAGCTTTCCCTCGTATAAATAATATGAGATTTTGATTATTAATCCCYTCTTTATTAATATTAATTTTAAGATCTTTAATTAATATTGGAGTAGGGACTGGTTGGACAGTTTATCCTCCKTTATCATTAAATATAAGTCATAGTGGAATATCTGTTGATTTGGCTATTTTTTCTTTACATATTGCGGGRGTTTCTTCTATTATAGGGGCAATAAATTTTATTACTACTATTTTAAATATRTGAATAATAAATATTAAAATTGATAAAATGTCTTTATTAATTTGATCAATTTTAATTACTGCTATTTTATTATTATTRTCTTTACCAGTTTTAGCAGGAGCTATTACTATATTATTAACAGATCGTAATTTAAATACAAGATTTTTTGATCCTTCYGGAGGGGGTGACCCAATTTTATATCAACATTTATTT

#### Morphological diagnosis.

Very similar to *E.zeirapherae*, differing by the characters given in the key as well as having the ovipositor sheath less setose. The COI barcodes of the two species differ by 6.29% (*p*-distance) all but ensuring that they are different species.

**Figure 8.**
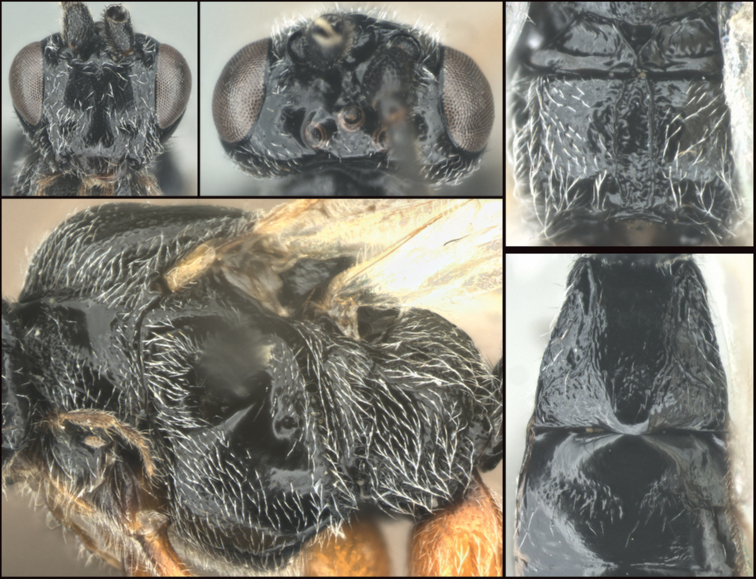
*Earinuswalleye* Sharkey, sp. nov., holotype.

#### Paratypes.

All are from the same locality as the holotype, 07PROBE-23096, 07PROBE-23097, 09PROBE-A0304. These are specimen IDs; more data on the specimens can be found by searching for these codes on BOLD (http://www.boldsystems.org).

**Figure 9.**
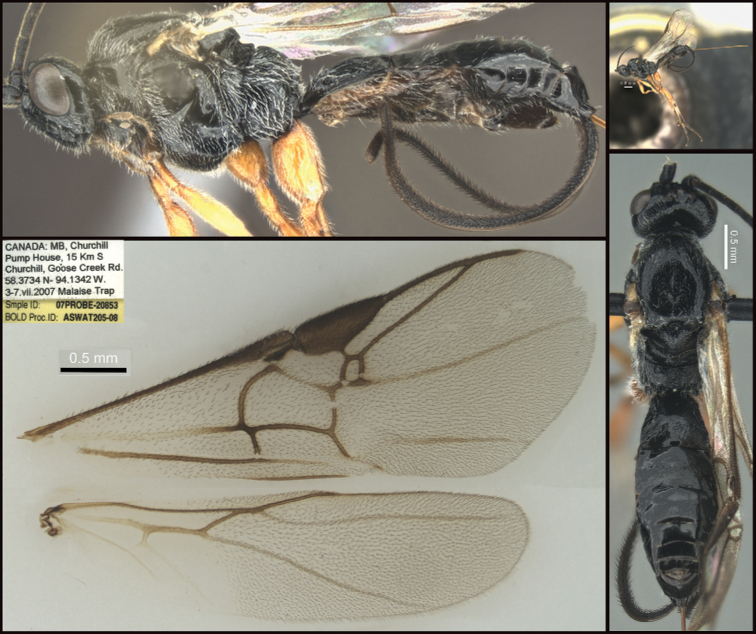
*Earinuswalleye* Sharkey, sp. nov., holotype.

#### Distribution.

Unknown but likely widespread in Alaska and northern and mid-latitudinal areas of Canada. Some or all records in Yu et al. (2016) for *E.zeirapherae* occurring from Alaska, Nunavut, and the Yukon may belong to this species.

#### Etymology.

Named in honor Stuart Walley (RIP), former research scientist at the Canadian National Collection and author of *E.zeirapherae*.

### ﻿ Earinus zeirapherae

Taxon classificationAnimaliaHymenopteraBraconidae

Walley, 1935

766B1C04-99FA-52A3-8733-9263AE5C92E1

Figs 10 , 11

#### Holotype.

♀, Grand River, Nova Scotia, 11.May.1932 (M. L. Prebble) No. 3847 (Canadian National Collection, viewed).

#### Biology.

The following are all reported as hosts by Yu et al. (2016). All belong to Tortricidae: *Aclerishudsoniana*, *Choristoneurarosaceana*, *Rhyacioniaadana*, *Zeirapheracanadensis*, *Zeirapheragriseana*, and *Zeirapheraratzeburgiana*. Since there are many species, including *E.austinbakeri* and *E.walleyi*, that are morphologically similar to *E.zeirapherae*, all hosts that do not belong to the genus *Zeiraphera* need confirmation.

#### Notes.

**Figure 10.**
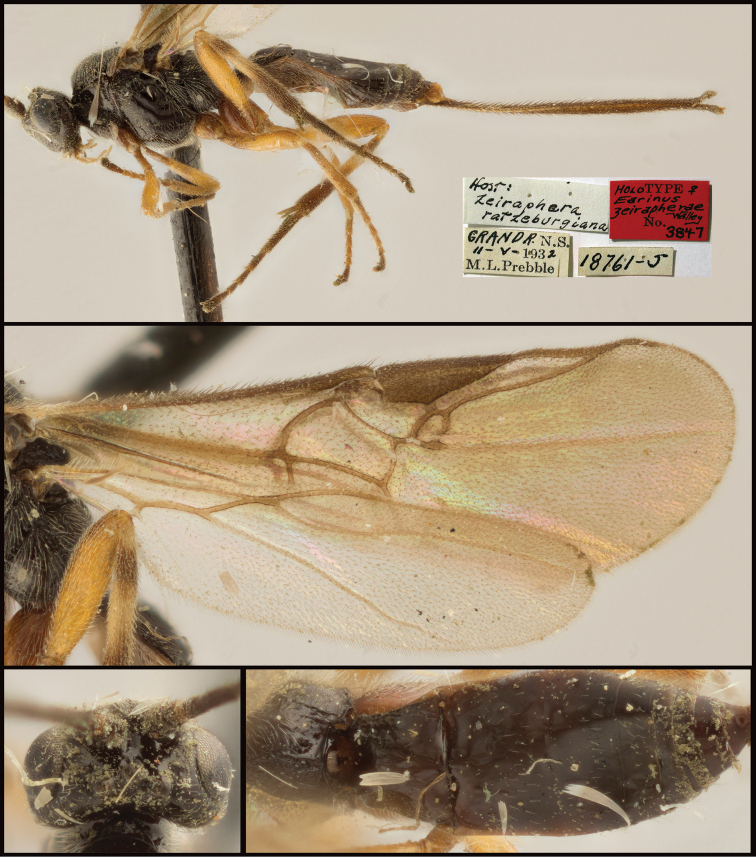
*Earinuszeirapherae*, holotype female.

**Figure 11.**
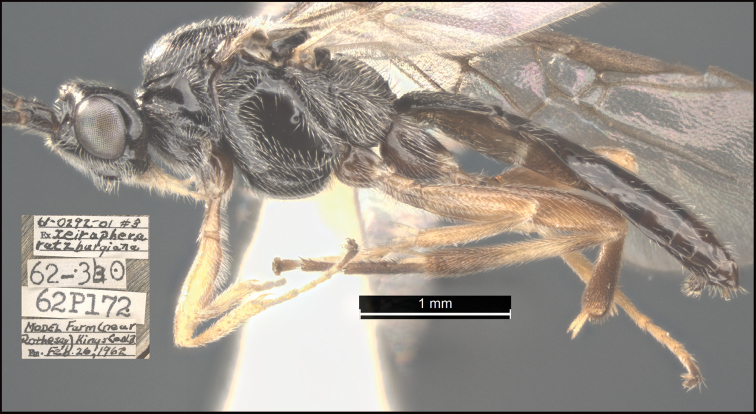
*Earinuszeirapherae*, male.

The holotype (Fig. 10) is from Nova Scotia, as is the male in Figure 11; both were reared from *Zeirapheraratzburgiana*. Contrary to the image of the holotype in Figure 10, the original description by Walley (1935) states that the fore and mid coxae and hind coxa are basally blackish, “front and middle coxae mostly, all trochanters faintly, hind coxae basally … blackish.” (Walley 1935: 56). It seems likely that over time the coxae of the holotype have faded. There are other specimens in the Canadian National Collection that have similar coloration but that are not likely to be conspecific based on other characters, e.g., one specimen from New Mexico. This serves as a reminder that the key will only function to separate the described species from each other.

### ﻿Key to the New World genera of Agathidinae

(Modified from Sharkey et al. 2021)

**Table:** 

1	**A** Forewing venation greatly reduced; RS absent and crossvein r present only as a short stub; Neotropical, rare	***Mesocoelus***
–	**B** Forewing venation moderately reduced; apical abscissa of RS absent, or mostly so, but crossvein r complete to junction of RS; Neotropical and rare	**2**
–	**C** Forewing venation not significantly reduced; apical abscissa of RS complete or almost complete to wing margin; widespread, common (99+ % of specimens)	**4**
	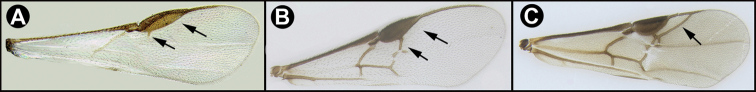	
2(1)	**A** Hind wing subbasal (SB) cell 4-sided with vein Cub emanating from an angle in the cell AND/OR **AA** Posterior surface of scutellum with a semi-circular or arc-shaped depression (post-scutellar depression)	***Therophilus* (in part)**
–	**B** Hind wing subbasal (SB) cell 3-sided. If Cub vein is present, it emanates from a straight vein. **BB** Post scutellar depression absent, but rugose sculpture usually present	**3**
	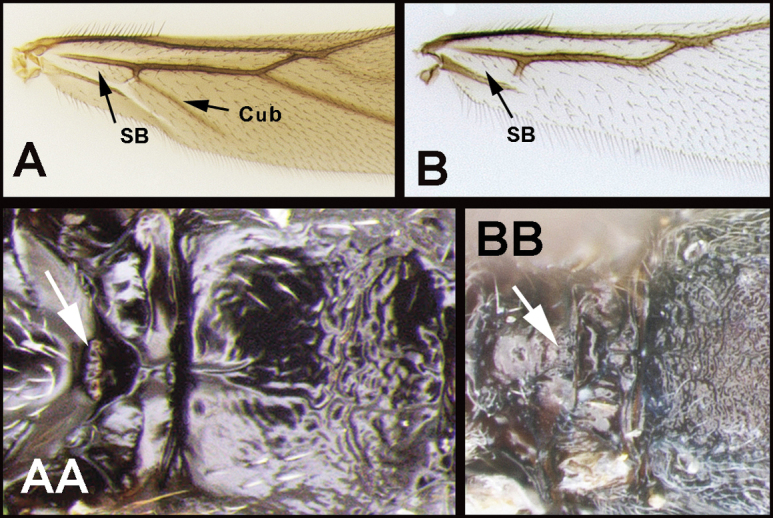	
3(2)	**A** Median area of first tergum not raised above lateral portions and granulate or striogranulate. **AA** Hind coxal cavities (HCC) open to metasomal foramen or narrowly closed and positioned partly above ventral margin of metasomal foramen (MF)	***Plesiocoelus***
–	**B** Median area of first tergum raised above lateral portions, sculpture variable but often smooth or smoothly striate. **BB** Hind coxal cavities closed and positioned completely below the metasomal foramen; ventral margin of metasomal foramen with a strong, relatively straight transverse carina (TC)	***Aerophilus* (in part)**
	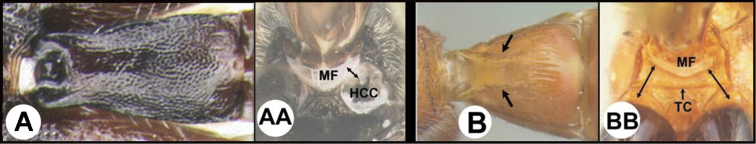	
4(1)	**A** Fore tarsal claws bifid	**5**
–	**B** Fore tarsal claws simple, with distinct basal lobe	**9**
–	**C** Fore tarsal claws simple, lacking a distinct basal lobe	**31**
	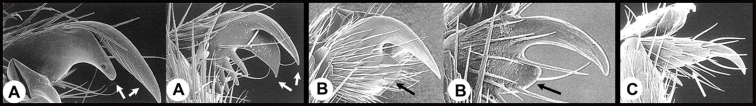	
5(4)	**A** Forewing areolet quadrate, not or only slightly narrower anteriorly. **AA** Ovipositor as long as or longer than half the length of metasoma	**7**
–	**B** Forewing areolet triangular or if quadrate much narrower anteriorly. **BB** Ovipositor shorter than half the length of metasoma	**6**
	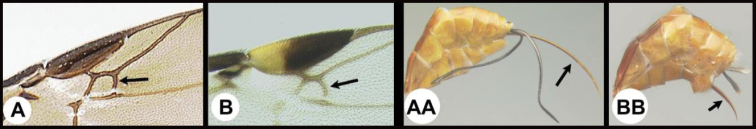	
6(5)	**A** Gena expanded into a flange posteriorly; malar space (MS) >½ length of eye height (EH); Neotropical, rare	***Hemichoma***
–	**B** Gena not modified into a flange posteriorly; malar space (MS) <½ length of eye height (EH); widespread, common	***Zelomorpha***
	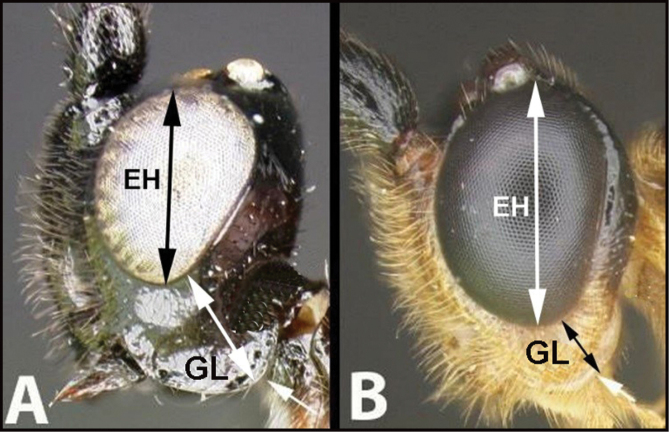	
7(5)	**A** Body predominantly orange/yellow. **AA** Frons bordered by a carina posteriorly; widespread, common	**8**
–	**B** Body predominantly black. **BB** Frons not bordered by a carina posteriorly; southern USA through the tropical Neotropics	***Zacremnops***
	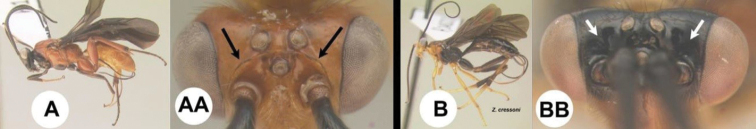	
8(7)	**A** Propodeum and hind coxa with granulate sculpture; first metasomal tergum almost 3× wider at apex than at base; rare; Neotropical, rare	***Labagathis***
–	**B** Propodeum and hind coxa lacking granulate sculpture; first metasomal tergum not nearly 3× wider at apex than at base; common; widespread, relatively common	***Cremnops***
	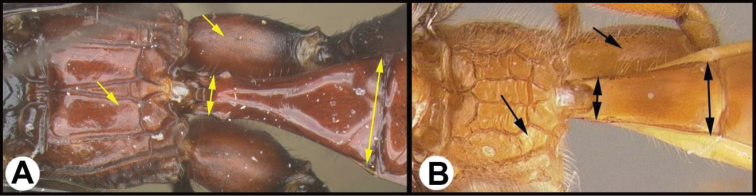	
9(4)	**A** Notauli present, though sometimes weak	**10**
–	**B** Notauli completely absent	**25**
	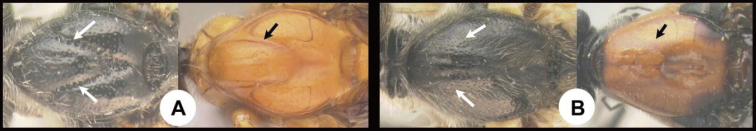	
10(9)	**A** Ventral margin of clypeus projecting; width of temple longer than width of eye in lateral view; Nearctic, rare	***Gelastagathis***
–	**B** Ventral margin of clypeus not projecting; width of temple shorter than width of eye in lateral view; widespread, common	**11**
	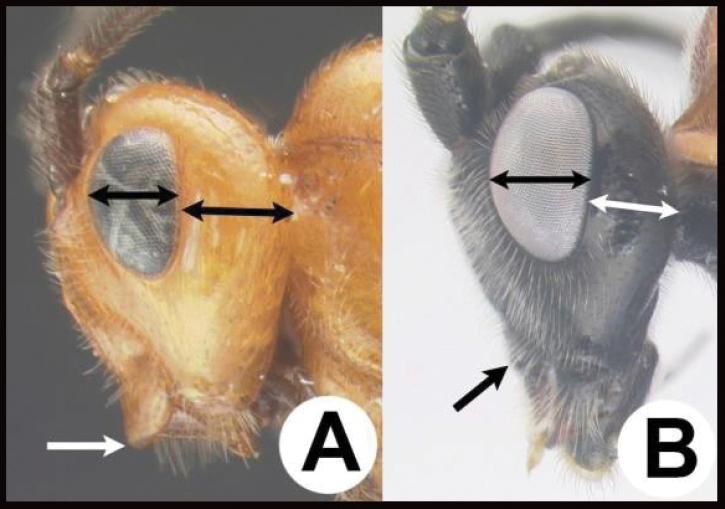	
11(10)	**A** Frons bordered by carinae or grooves posteriorly	**12**
–	**B** Frons not bordered by carinae or grooves posteriorly	**14**
	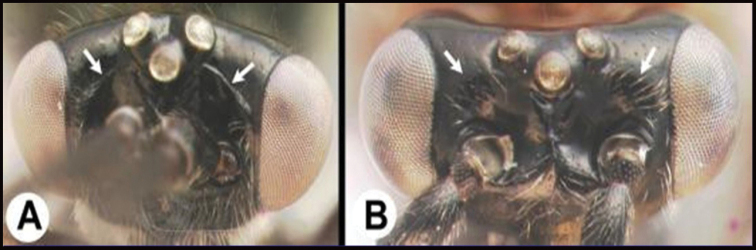	
12(11)	**A** Hind coxa with granulate sculpture. **AA** Second submarginal cell minute or absent; Neotropical, rare	***Trachagathis***
–	**B** Hind coxa smooth, lacking granulate sculpture. **BB** Second submarginal cell of normal dimensions; widespread, relatively common	**13**
	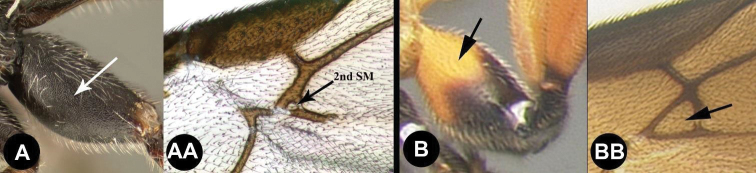	
13(12)	**A** First metasomal tergum smooth with two widely spaced converging carinae forming a tear-shaped basal area; Neotropical, rare	***Pharpa***
–	**B** First metasomal tergum usually smooth and convex, or **BB** with a median longitudinal carina, or, **BBB** rarely with 2 carinae in which case the tergum has more extensive sculpture; widespread, common	***Alabagrus***
	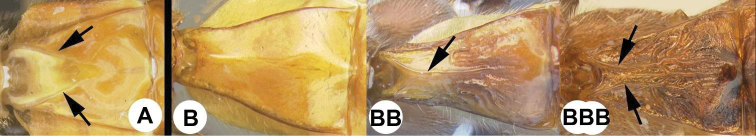	
14(11)	**A** Malar space (MS) distinctly >½ eye height (EH). Head shape in frontal view elongate, at least as high (measure from ventral margin of clypeus) as wide	**15**
–	**B** Malar distance usually (95%) ≤½ eye height. Head shape in frontal view wide, wider than high (measure from ventral margin of clypeus)	**16**
	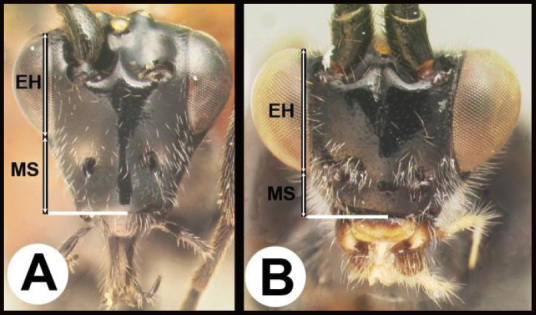	
15(14)	**A** Third tergum completely smooth; pair of carinae on first tergum not prominent. **AA** Hind coxal cavities (HCC) open to metasomal foramen or narrowly closed and positioned partly above ventral margin of metasomal foramen (MF); common in the Nearctic, very rare in the Neotropics	***Agathis* (in part)**
–	**B** Third tergum usually (95%) partly or completely sculptured, often sculpture confined to narrow line along transverse depression; pair of carinae on first tergum prominent. **BB** Hind coxal cavities closed and positioned completely below the metasomal foramen; ventral margin of metasomal foramen with a strong, relatively straight transverse carina (TC); widespread, common	***Aerophilus* (in part)**
	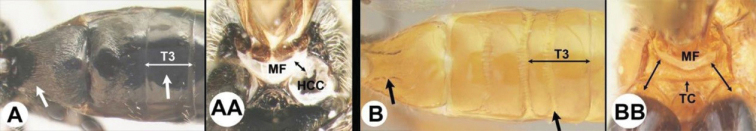	
16(14)	**A** Propodeal spiracle elongate, > 2× longer than wide; widespread, common	***Pneumagathis***
–	**B** Propodeal spiracle circular or oval, < 2× longer than wide	**17**
	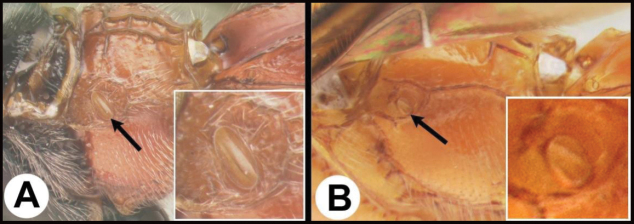	
17(16)	**A** Pair of carinae on first tergum NOT prominent. **AA** Hind coxal cavities (HCC) open to metasomal foramen or narrowly closed and positioned partly above ventral margin of metasomal foramen (MF)	**18**
–	**B** Pair of carinae on first tergum prominent; **BB** Hind coxal cavities closed and positioned below the metasomal foramen; ventral margin of metasomal foramen with a strong, relatively straight transverse carina (TC); widespread, common	***Aerophilus* (in part**)
	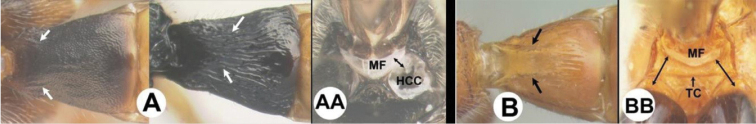	
18(15)	**A** First tergum completely smooth, or rarely with some punctures posterolaterally	**20**
–	**B** First tergum with sculpture	**19**
	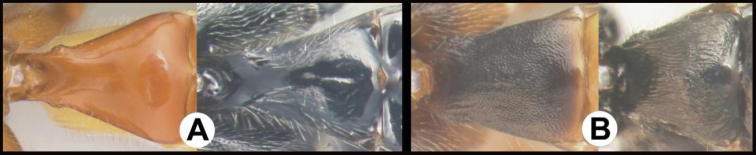	
19(18)	**A** Cub vein of hind wing long and partly tubular, apical margin of subbasal (SB) cell angled; widespread, common	***Therophilus* (in part)**
–	**B** Cub vein of hind wing weak or absent and never tubular; apical margin of subbasal cell (SB) straight. Nearctic and northern Neotropics, i.e., Mexico and Central America, rare	***Agathirsia* (in part)**
	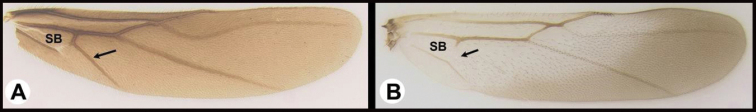	
20(18)	**A** Notauli pitted or crenulate	**21**
–	**B** Notauli smooth	**24**
	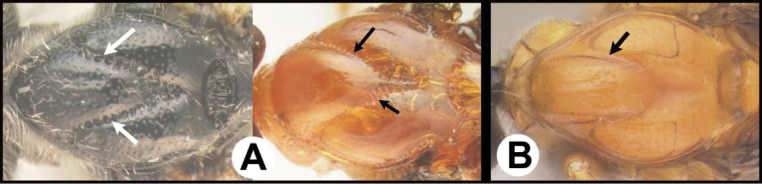	
21(17)	**A** Third tergum usually entirely smooth or weakly and partly coriarious (leather-like), if with different sculpture (especially in transverse depressions) then pair of longitudinal carinae on first metasomal tergum weaker than in B or absent. **AA** Hind coxal cavities (HCC) open to metasomal foramen or narrowly closed and positioned partly above ventral margin of metasomal foramen (MF)	**22**
–	**B** Third tergum usually partly or completely sculptured, often sculpture confined to narrow line along transverse depression. **B** Pair of longitudinal carinae on first metasomal tergum present and extending past spiracles. **BB** Hind coxal cavities closed and positioned entirely below the metasomal foramen (MF); ventral margin of metasomal foramen with a strong, relatively straight transverse carina (TC); widespread, common	***Aerophilus* (in part)**
	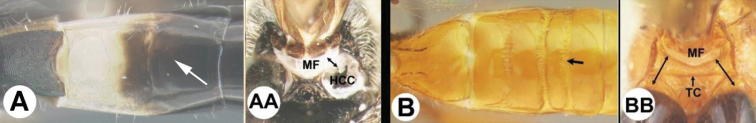	
22(21)	**A** First tergum partly or completely granulate; widespread, common	***Neothlipsis***
–	**B** First tergum otherwise sculptured, usually striate or rugosostriate	**23**
	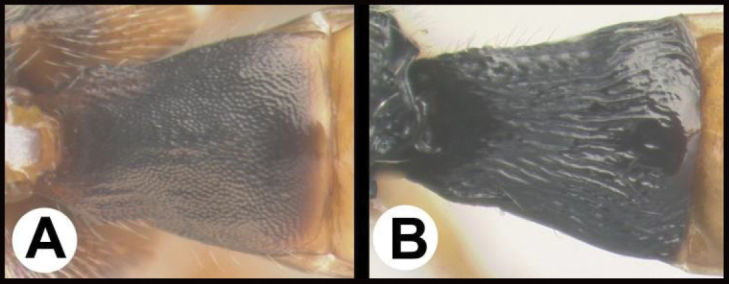	
23(22)	**A** Posterior apex of scutellum with a distinct depression in the form of a semicircle or two distinct pits; widespread, common	***Therophilus* (in part)**
–	**B** Posterior apex of scutellum lacking depression, smooth to rugose; common in the Nearctic, very rare in the Neotropics	***Agathis* (in part)**
	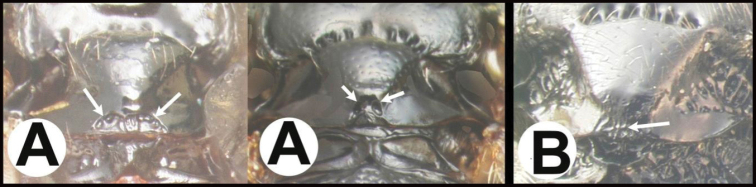	
24(19)	**A** Propleuron with a distinct protuberance; gena expanded into an acute angle posteroventrally; Neotropical, rare	***Zamicrodus***
–	**B** Propleuron flat or weakly convex, lacking a distinct protuberance; genae not expanded and rounded posteroventrally; Neotropical, rare	***Aphelagathis***
	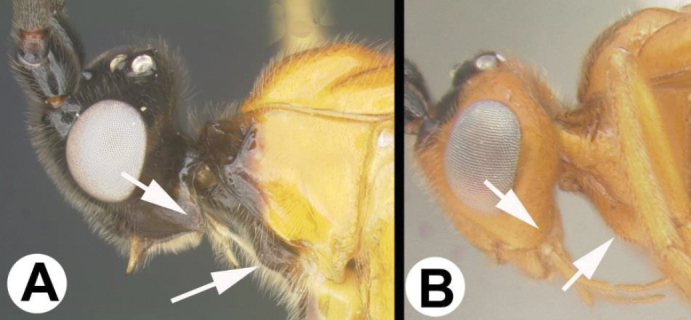	
25(9)	**A** Second submarginal cell of forewing quadrate. **AA** Cub vein of hind wing present and often tubular; subbasal cell angled distally at junction of Cub	**26**
–	**B** Not combining the above character states. **B.** Second submarginal cell usually triangular. **BB** Cub vein of hind wing usually absent or not tubular and subbasal cell not angled distally; widespread, common	**27**
	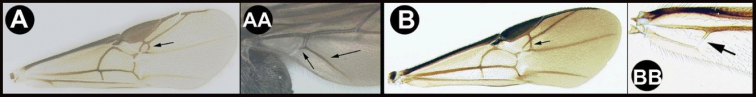	
26(25)	**A** Face distinctly punctate **AA.** Body and antenna black, all femora pale (yellow to orange), hind tibia pale (yellow to orange with a melanic apex and a melanic ring or lateral spot subbasally. **AAA** Holarctic and Oriental (in the Americas from northern Canada and rarely as far south as northern Mexico)	***Earinus***
–	**B** Face smooth with very tiny punctation **BB** Often brightly colored and otherwise not as above. **BBB** Neotropical: Chile, and southern Argentina, and rarely in high altitudes of the Andes far north as Colombia and Ecuador	***Chilearinus***
	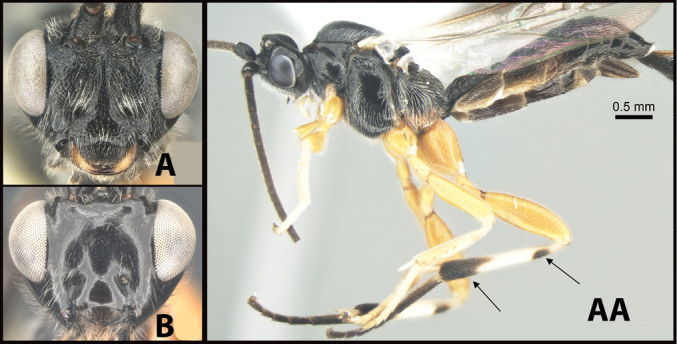	
27(25)	**A** Third tergum completely smooth. **AA** Hind coxal cavities (HCC) open to metasomal foramen (MF), or narrowly closed such that the ventral part of the metasomal foramen is below the dorsal margin of the hind coxal cavities	**28**
–	**B** Third tergum usually (95% of specimens encountered) partly or completely sculptured, often sculpture confined to narrow lines along transverse depressions. **BB** Hind coxal cavities closed and positioned completely below the metasomal foramen; ventral margin of metasomal foramen with a strong, relatively straight transverse carina; widespread, common	***Aerophilus* (in part**)
	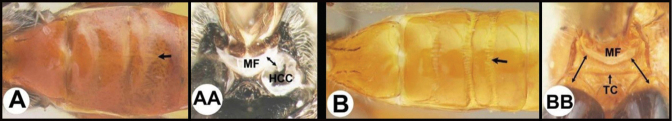	
28(27)	**A** Spurious vein, RS2b, well developed. **AA** Ovipositor barely exerted, much shorter than metasoma; Neotropical, very rare	***Marjoriella***
–	**B** Spurious vein, RS2b, lacking. **BB** Ovipositor at least as long as metasoma	**29**
	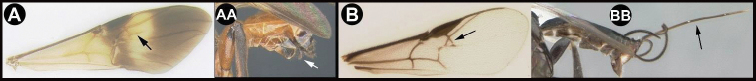	
29(28)	**A** Second submarginal cell smaller than its dorsal stem; apical abscissa of RS curving towards fore margin of wing; Neotropical, very rare	***Smithagathis***
–	**B** Second submarginal cell larger than its dorsal stem; apical abscissa of RS straight	**30**
	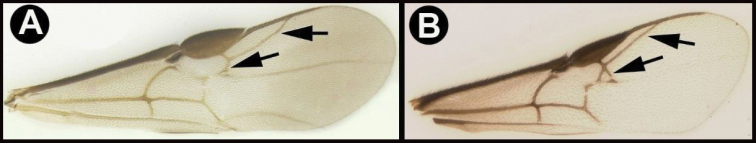	
30(29)	**A** Posterolateral corner of gena sharp; propleuron with a protuberance; Neotropical, rare	***Amputoearinus***
–	**B** Posterolateral corner of gena rounded; propleuron evenly convex, lacking a protuberance; widespread, common	***Lytopylus***
	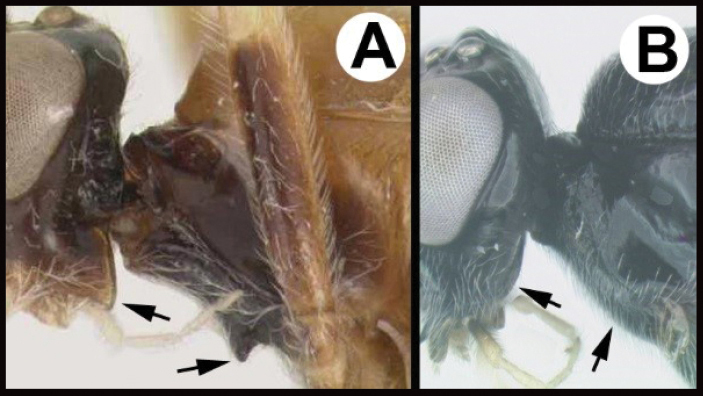	
31(4)	**A** Notauli absent, mesoscutum completely smooth; Neotropical, rare	***Sesioctonus***
–	**B** Notauli present though sometimes only indicated anteriorly or posteriorly; widespread and common in the Nearctic, rare in the Neotropics	**32**
	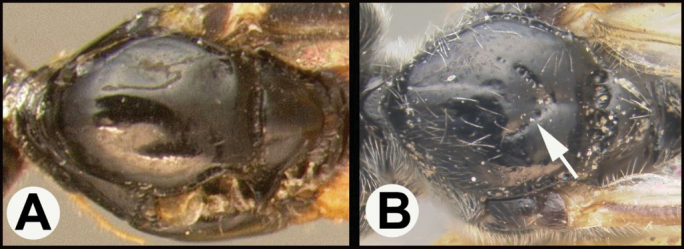	
32(31)	**A** First tergum smooth, lacking microsculpture and pair of longitudinal carinae, at most with punctures laterally	**33**
–	**B** First tergum with microsculpture, usually in the form of longitudinal striae or rugae; widespread and common in the Nearctic, extremely rare in the Neotropics	***Agathis* (in part)**
	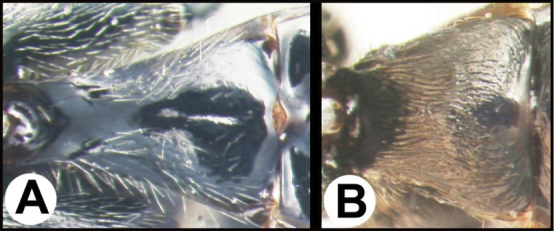	
33(32)	**A** Ovipositor barely exerted, shorter than half the length of metasoma; Nearctic and Central America, rare	***Crassomicrodus***
–	**B** Ovipositor at least as long as half the metasoma, often much longer; Nearctic and Central America, rare	***Agathirsia* (in part)**
	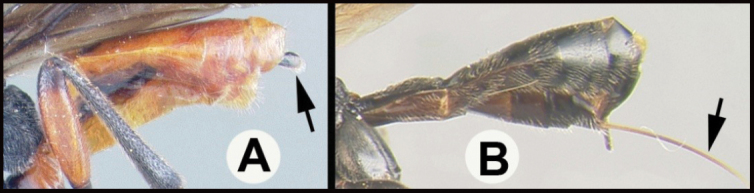	

## Supplementary Material

XML Treatment for Chilearinus

XML Treatment for Chilearinus covidchronos

XML Treatment for Chilearinus janbert

XML Treatment for Earinus

XML Treatment for Earinus austinbakeri

XML Treatment for Earinus erythropoda

XML Treatment for Earinus limitaris

XML Treatment for Earinus walleyi

XML Treatment for Earinus zeirapherae
